# Sufentanil Sublingual Tablet System vs. Intravenous Patient-Controlled Analgesia with Morphine: Postoperative Pain Control and Its Impact in Quality of Recovery

**DOI:** 10.7759/cureus.47302

**Published:** 2023-10-19

**Authors:** Diana L Fernandes, Ana Isabel G Pereira, Ana Amorim, Joana Freitas

**Affiliations:** 1 Anaesthesiology, Funchal Central Hospital, Funchal, PRT

**Keywords:** morphine, sufentanil, patient-controlled, analgesia, postoperative pain

## Abstract

Context: Patient-controlled analgesia (PCA) is commonly used for postoperative pain control. Although widely used, intravenous (IV) morphine PCA may not be suitable for all patients. Sufentanil sublingual tablet system (SSTS) PCA is a recent technique that has had success as a safe and effective alternative for acute pain management.

Aims: This study aims to compare both the efficacy and safety of SSTS PCA versus IV morphine PCA in postoperative pain control and the quality of recovery in adult patients following scheduled gynecological or orthopedic surgery.

Settings and design: Open-label, parallel-group, randomized controlled trial with 54 patients. The primary outcome was postoperative pain control, while the secondary outcomes included adverse effects associated with two analgesic modalities, total opioid dose required, patient satisfaction, and impact on the quality of postoperative recovery.

Methods and material: Statistical analysis was performed using IBM SPSS Statistics for Windows, Version 26.0 (Released 2019; IBM Corp., Armonk, New York, United States). The chi-squared test was used in categorical variables. When distribution was normal, T-student (mean ± standard deviation) was used in continuous variables. In contrast, when distribution was not normal, the Mann-Whitney test (median (minimal-maximal)) was used.

Results: The results showed that there was a statistically significant difference in the total dose of opioid used by patients at 24 hours postoperatively, with patients receiving SSTS PCA requiring a higher total dose when compared to those receiving IV morphine PCA. However, there were no statistically significant differences in pain scores, adverse events, or patient satisfaction.

Conclusions: The study suggests that both IV morphine and sublingual sufentanil are safe and effective for postoperative pain management.

## Introduction

Patient-controlled analgesia (PCA) is a widespread and frequently used pain management method in hospitalized patients. Intravenous (IV) morphine PCA is commonly used in postoperative pain control, but it requires IV access and can lead to associated complications [1-3]. Sufentanil sublingual tablet system (SSTS) PCA is a newer technique that allows patients to self-administer sublingual sufentanil using a handheld device, which requires no IV access [4-7]. This study aimed to compare the efficacy and safety of SSTS PCA and IV morphine PCA in postoperative pain control and the quality of recovery in adult patients following scheduled gynecological or orthopedic surgery.

## Materials and methods

**Figure 1:**
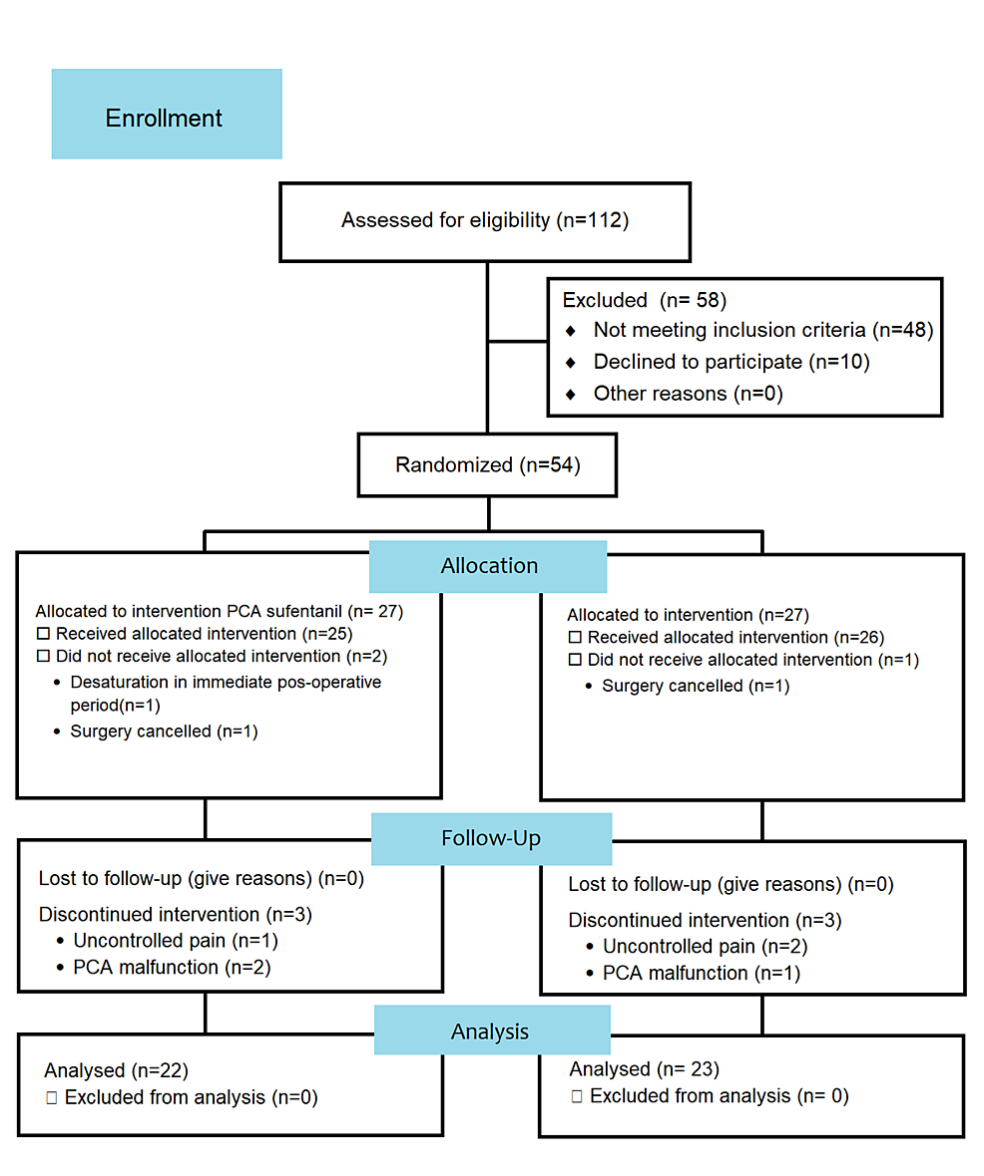
2010 CONSORT flow diagram CONSORT: Consolidated Standards of Reporting Trails

The study was a single-center, open-label, parallel-group, randomized controlled trial and followed the 2010 Consolidated Standards of Reporting Trails (CONSORT) statement guidelines (Figure 1). 

The sample size calculation for this study was based on several factors. First, there is a positive correlation between the visual analogue scale (VAS) and numeric rating scale (NRS) [8-10]. Second, the study aims to detect a minimal clinically important difference of 1 in NRS in a postoperative setting [11]. Third, a two-sided significance level (alpha) of 0.05 was chosen. Finally, the researchers expected a 35% drop off rate and changes in analgesic prescription after the evaluation of an acute pain physician. Based on these factors, the researchers determined that a total sample size of 54 patients would be appropriate for the study.

The study was approved by the Funchal Central Hospital Ethics Committee (approval number: 35/2019) and registered in ClinicalTrials.gov (ID NCT05259098). The study was conducted in Funchal Hospital and data was collected between November 2019 and June 2020. The inclusion criteria were signed informed consent, 18+ years, physical status according to the American Society of Anesthesiologists (ASA) 1-3, and undergoing scheduled open hysterectomy, total knee arthroplasty, or total hip arthroplasty, while the exclusion criteria were refusal to participate, not of legal age or legally dependent of thirds, neurological or psychiatric pathologies or an altered state of consciousness that does not allow for the PCA strategy, documented drinking habits and/or consumption of illicit drugs, diagnosis of opioid use disorder, tolerance to opioid therapy (use of > 15 mg oral morphine or equivalent, per day, in the past three months), obstructive sleep apnea syndrome (OSAS) documented, patients on long-term oxygen therapy, intraoperative use of intrathecal morphine, and current usage of any anesthetic techniques that provide postoperative analgesia (epidural catheter, peripheral nerve blocks, or infiltration of the surgical wound with local anesthetic). Data was collected in the anesthesia department and at the bedside of patients, while medical records were assessed in the institutional information system. A permuted block randomization with 1:1 allocation ratio was performed using an online software. A total of 10 blocks were created, consisting of seven blocks with six and three blocks with four participants.

The primary outcome was postoperative pain control measured at 24 hours and 48 hours using the NRS. In contrast, the secondary outcome assessed adverse effects associated with both analgesic modalities measured every eight hours by the nursing team (sedation, hypoxemia, nausea, vomiting, pruritus, and urinary retention), required dosage of opioid in each strategy, patients' satisfaction, and the impact of the treatment on the quality of postoperative recovery using the validated Portuguese version of the Quality of Recovery-15 (QoR-15) questionnaire [12].

Preemptive analgesia included the use of oral gabapentin (100 mg) administered one hour before the procedure, and IV ketorolac (30 mg) at the beginning of the surgery. Intraoperatively, nociception was controlled with fentanyl in boluses. At the end of the procedure, 1 g of IV paracetamol was administered. In the postoperative period, patients were admitted to the post-anesthetic care unit (PACU) for initial recovery. While there, they received comprehensive instructions on the appropriate use of PCA devices. It was not necessary to administer any bolus in the PACU by the nursing staff, as the pain was under control, allowing the patient to be taught how the machine works. The morphine PCA was Smiths Medical CADD Model 2020® (Smiths Medical, Minneapolis, Minnesota, United States), programmed 1 mcg IV morphine on demand, 10 minutes lockout, maximum 6 mcg per hour, while the SSTS was Zalviso® machine (AcelRx Pharmaceuticals, San Mateo, California, United States), programmed 15 mg sublingual sufentanil, 20 minutes lockout, maximum 45 mg per hour. All patients were prescribed a combination of analgesics that included both IV paracetamol (1 g every eight hours), IV non-steroidal anti-inflammatory drugs (ketorolac 30 mg every eight hours), and oral gabapentin (100 mg every eight hours, initiated one hour before the procedure). All patients had also prescribed a fixed antiemetic medication (IV ondansetron, 4 mg every eight hours).

Statistical analysis was performed using IBM SPSS Statistics for Windows, Version 26.0 (Released 2019; IBM Corp., Armonk, New York, United States). The chi-squared test was used in categorical variables. When distribution was normal, T-student (mean ± standard deviation) was used in continuous variables. In contrast, when distribution was not normal, the Mann-Whitney test (median (minimal-maximal)) was used.

## Results

Statistical analysis was performed to compare the effectiveness of IV morphine PCA with SSTS PCA in postoperative pain management. There was no difference in demographic characteristics between the two groups (Table 1).

**Table 1:** Sample baseline demographic and clinical characteristics PCA: patient-controlled analgesia; ASA: American Society of Anesthesiologists The chi-squared test was used in categorical variables. When distribution was normal, T-student (mean ± standard deviation) was used in continuous variables. In contrast, when distribution was not normal, the Mann-Whitney test (median (minimal-maximal)) was used

Variable	PCA			p-value
	Total (n = 45)	Morphine (n = 23)	Sufentanil (n = 22)	
Age (in years)	58.98 ± 11.80	60.78 ± 11.24	57.09 ± 12.32	0.299
Female (%)	40 (88.9)	20 (87.0)	20 (90.9)	0.673
Body mass index	30 (18-44)	30 (21-38)	30 (18-44)	0.837
ASA (%)				
1	5 (11.1)	2 (8.7)	3 (13.6)	0.686
2	37 (82.2)	20 (87.0)	17 (77.3)	
3	3 (6.7)	1 (4.3)	2 (9.1)	
Surgery (%)				
Total hip arthroplasty	6 (13.3)	3 (13.0)	3 (13.6)	0.539
Total knee arthroplasty	22 (48.9)	13 (56.5)	9 (40.9)	
Hysterectomy	17 (37.8)	7 (30.4)	10 (45.5)	
Anesthesia technique (%)				
Balanced general anesthesia	18 (40.0)	7 (30.4)	11 (50.0)	0.181
Locoregional anesthesia	27 (60.0)	16 (69.6)	11 (50.0)	
Respiratory comorbidity (%)	6 (13.3)	3 (13.0)	3 (13.6)	0.953
Cardiovascular comorbidity (%)	23 (51.1)	13 (56.5)	10 (45.5)	0.458

With regard to the primary outcome, no statistically significant differences were identified in the pain scores between the two groups at point in time. The median of NRS at 24 hours at rest was 1 (0-8) for the morphine group and 1.5 (0-7) for the sufentanil group. At 24 hours postoperative, patients reported moderate pain during movement with a median score of 5 (2-9) and 5.5 (0-9) for morphine and sufentanil, respectively. At 48 hours postoperative, the mean pain score for both groups at rest was 0 and 3 during movement (Table 2).

**Table 2:** Pain control at 24 hours and 48 hours PCA: patient-controlled analgesia; NRS: numeric rating scale The Mann-Whitney test (median (minimal-maximal)) was used

Variable	PCA			p-value
	Total (n = 45)	Morphine (n = 23)	Sufentanil (n = 22)	
24-hour NRS score at rest	1 (0-8)	1 (0-8)	1.5 (0-7)	0.933
24-hour NRS score during movement	5 (0-9)	5 (2-9)	5.5 (0-9)	0.697
48-hour NRS score at rest	0 (0-5)	0 (0-4)	0 (0-5)	0.739
48-hour NRS score during movement	3 (0-9)	3 (0-7)	3 (0-9)	0.279

The data used in the analysis of the total dose of opioid required in each strategy was obtained from the pharmacokinetic properties of sublingual sufentanil, considering that 15 mg sublingual sufentanil is equal to 3 mg IV morphine. This conversion is based on 300-400 potency factor and 60% bioavailability of sublingual sufentanil [7]. Specifically, results showed that the dosage of medication at 24 hours postoperative used by patients who received SSTS was higher than that used by patients who received IV morphine (median difference 8.4 mg, p=0.040). However, there were no statistically significant differences in the incidence of adverse events, including nausea, vomiting, sedation, or respiratory depression. Patient satisfaction scores were also similar between the two groups. The 24-hour QoR-15 did not significantly differ between the groups (Table 3 and Table 4).

**Table 3:** Secondary outcomes at 24-hour evaluation PCA: patient-controlled analgesia; RASS: Richmond agitation-sedation scale; SpO2: oxygen saturation; QoR-15: Quality of Recovery-15 *This conversion is based in the pharmacokinetic properties of sublingual sufentanil which reported that 15 mg sublingual sufentanil is equal to 3 mg IV morphine (based on 300-400 potency factor and 60% bioavailability of sublingual sufentanil). The chi-squared test was used in categorical variables. The Mann-Whitney test (median (minimal-maximal)) was used in continuous variables

Variable	PCA			p-value
	Total (n = 45)	Morphine (n = 23)	Sufentanil (n = 22)	
Medication dosage at 24 hours in milligram of equipotency with morphine*	24.5 (3-75)	20.1 (8-69)	28.5 (3-75)	0.040
24-hour lateral effects (%)				
Sedation (RASS < -2)	0 (0)	0 (0)	0 (0)	-
Hypoxemia (SpO2 < 90%)	0 (0)	0 (0)	0 (0)	-
Nausea	8 (17.8)	2 (8.7)	6 (27.3)	0.103
Pruritus	3 (6.7)	2 (8.7)	1 (4.5)	0.577
Urinary retention	4 (8.9)	2 (8.7)	2 (9.1)	0.963
Vomiting	4 (8.9)	4 (17.4)	-	-
24-hour pain relief (%)				
Nothing	2 (4.4)	2 (8.7)	0 (0.0)	0.186
Little	5 (11.1)	2 (8.7)	3 (13.6)	
Mild	19 (42.2)	12 (52.2)	7 (31.8)	
Much	19 (42.2)	7 (30.4)	12 (54.5)	
24-hour global satisfaction (%)				
Little	4 (8.9)	3 (13.0)	1 (4.5)	0.237
Enough	3 (6.7)	1 (4.3)	2 (9.1)	
Good	25 (55.6)	15 (65.2)	10 (45.5)	
Great	13 (28.9)	4 (17.4)	9 (40.9)	
24-hour sum of QoR-15 score	118 (65-206)	112 (65-136)	124.5 (83-206)	0.061

**Table 4:** Secondary outcomes at 48-hour evaluation PCA: patient-controlled analgesia; RASS: Richmond agitation-sedation scale; SpO2: oxygen saturation *This conversion is based in the pharmacokinetic properties of sublingual sufentanil which reported that 15 mg sublingual sufentanil is equal to 3 mg IV morphine (based on 300-400 potency factor and 60% bioavailability of sublingual sufentanil) **One patient missing data about these two variables. The chi-squared test was used in categorical variables. The Mann-Whitney test (median (minimal-maximal)) was used in continuous variables

Variable	PCA			p-value
	Total (n = 45)	Morphine (n = 23)	Sufentanil (n = 22)	
Medication dosage at 48 hours in milligram of equipotency with morphine*	15.6 (3-117)	15.6 (6-43.1)	16.5 (3-117)	0.670
48-hour lateral effects (%)				
Sedation (RASS < -2)	0 (0)	0 (0)	0 (0)	-
Hypoxemia (SpO2 < 90%)	0 (0)	0 (0)	0 (0)	-
Nausea	8 (17.8)	3 (13.0)	5 (22.7)	0.396
Pruritus	0 (0)	0 (0)	0 (0)	-
Urinary retention	0 (0)	0 (0)	0 (0)	-
Vomiting	0 (0)	0 (0)	0 (0)	-
48-hour pain relief (%)**				
Mild	20 (45.5)	10 (45.5)	10 (45.5)	1.000
Much	24 (54.5)	12 (54.5)	12 (54.5)	
48-hour global satisfaction (%)**				
Enough	2 (4.5)	0 (0)	2 (9.1)	0.236
Good	24 (54.5)	14 (63.6)	10 (45.5)	
Great	18 (40.9)	8 (36.4)	10 (45.5)	

## Discussion

The present study aimed to compare the effectiveness of IV morphine PCA with SSTS PCA for postoperative pain management. Results indicated that both methods were equally effective in providing pain relief, which is consistent with previous studies that have reported similar findings [4,7]. Since both PCA methods allow patients to self-administer pain medication based on their pain levels, the results were expected to be comparable. In our point of view, a PCA system that does not involve an IV access may be advantageous in some patients, in the sense of enabling a rapid recovery in an Enhanced Recovery After Surgery (ERAS) program.

However, the study revealed a significant difference in the total amount of opioids used by patients receiving sublingual sufentanil PCA when compared to those receiving IV morphine PCA, with the former requiring a higher total dose at 24 hours postoperatively. This could be either due to differences in pharmacokinetics and individual variations in pain response or because the conversion factor may not apply ubiquitously to any patient or conditions. One possibility that can also explain this fact is that the maximum dose per hour is higher in the group allocated to sufentanil (45 mcg, equivalent to 9 mg of morphine per hour) compared to that of morphine (6 mg per hour). Despite the difference, we wanted to test these treatment regimens since they are the ones that are protocolled in our department. Regardless of the difference in dosage, the side effects (which can be dose dependent) were similar between both groups, confirming findings of other studies [4,7].

Although there was no statistically significant difference in pain relief and patient satisfaction between the two groups, the small sample size could have affected the results.

Generalizations of this study are limited by the specific inclusion and exclusion criteria, which targeted patients undergoing scheduled hysterectomy, total knee arthroplasty, or total hip arthroplasty and excluded patients with certain medical conditions or using certain analgesic techniques. Additionally, the study was conducted at a single institution, which may limit the generalizability of the findings to other settings.

A strong point of the study is the following of the CONSORT guidelines, recognized standards for reporting randomized controlled trials. Additionally, the study used validated outcome measures, and the sample size calculation was based on an a priori power analysis.

However, the study has some limitations. With regard to the exclusion criteria, we considered a dose to exclude patients with chronic opioid use; however, we did not consider the duration of prior use. It was an open-label study, which means that both the patients and clinicians were aware of the treatment allocation, and this may have introduced bias. Additionally, the study did not assess long-term outcomes beyond the first 48 hours after surgery. 

Another concern is that sufentanil is more lipophilic, is more potent, and has a faster onset of action than morphine, which raises the question of it being associated with a higher risk of addiction.

## Conclusions

This study suggests that both IV morphine and sublingual sufentanil are effective and safe options for postoperative pain management. However, clinicians should consider the total dose required to achieve pain relief when choosing between the two medications. Future research could explore other factors that may influence the effectiveness and safety of these medications in specific patient populations. However, further studies with larger sample sizes are needed to confirm these findings.

## References

[1] Mann C, Ouro-Bang'na F, Eledjam JJ (2005). Patient-controlled analgesia. Curr Drug Targets.

[2] Momeni M, Crucitti M, De Kock M (2006). Patient-controlled analgesia in the management of postoperative pain. Drugs.

[3] Ilias W, le Polain B, Buchser E, Demartini L (2008). Patient-controlled analgesia in chronic pain patients: experience with a new device designed to be used with implanted programmable pumps. Pain Pract.

[4] Frampton JE (2016). Sublingual sufentanil: a review in acute postoperative pain. Drugs.

[5] Minkowitz HS, Leiman D, Melson T, Singla N, DiDonato KP, Palmer PP (2017). Sufentanil sublingual tablet 30 mcg for the management of pain following abdominal surgery: a randomized, placebo-controlled, phase-3 study. Pain Pract.

[6] Ringold FG, Minkowitz HS, Gan TJ, Aqua KA, Chiang YK, Evashenk MA, Palmer PP (2015). Sufentanil sublingual tablet system for the management of postoperative pain following open abdominal surgery: a randomized, placebo-controlled study. Reg Anesth Pain Med.

[7] Melson TI, Boyer DL, Minkowitz HS, Turan A, Chiang YK, Evashenk MA, Palmer PP (2014). Sufentanil sublingual tablet system vs. intravenous patient-controlled analgesia with morphine for postoperative pain control: a randomized, active-comparator trial. Pain Pract.

[8] Bijur PE, Latimer CT, Gallagher EJ (2003). Validation of a verbally administered numerical rating scale of acute pain for use in the emergency department. Acad Emerg Med.

[9] Breivik H, Borchgrevink PC, Allen SM (2008). Assessment of pain. Br J Anaesth.

[10] Kelly AM (2001). The minimum clinically significant difference in visual analogue scale pain score does not differ with severity of pain. Emerg Med J.

[11] Myles PS, Myles DB, Galagher W, Boyd D, Chew C, MacDonald N, Dennis A (2017). Measuring acute postoperative pain using the visual analog scale: the minimal clinically important difference and patient acceptable symptom state. Br J Anaesth.

[12] Sá AC, Sousa G, Santos A, Santos C, Abelha FJ (2015). Quality of recovery after anesthesia: validation of the Portuguese version of the 'quality of recovery 15' questionnaire. Acta Med Port.

